# Cardamom Essential Oil Exerts a Curative Effect Against Kiwifruit Bacterial Canker but Fails to Activate Host Defense Mechanisms

**DOI:** 10.3390/plants15162533

**Published:** 2026-08-21

**Authors:** Miguel G. Santos, Marta Nunes da Silva, Tânia R. Fernandes, Andreia Garrido, Nuno Mariz-Ponte, Marta W. Vasconcelos, Susana M. P. Carvalho

**Affiliations:** 1 GreenUPorto—Sustainable Agrifood Production Research Centre/Inov4Agro, DGAOT, Faculty of Sciences, University of Porto, Campus de Vairão, Rua da Agrária 747, 4485-646 Vila do Conde, Portugal; mgsantos@fc.up.pt (M.G.S.); mansilva@ucp.pt (M.N.d.S.); tania.fernandes@fc.up.pt (T.R.F.); andreia.garrido@fc.up.pt (A.G.); nuno.ponte@fc.up.pt (N.M.-P.); 2 CBQF—Centro de Biotecnologia e Química Fina—Laboratório Associado, Escola Superior de Biotecnologia, Universidade Católica Portuguesa, Rua Diogo Botelho 1327, 4169-005 Porto, Portugal; mvasconcelos@ucp.pt

**Keywords:** *Actinidia chinensis*, antimicrobial activity, gene expression, *Elettaria cardamomum*, *Pseudomonas syringae* pv. *actinidiae*, ROS homeostasis, sustainable disease control

## Abstract

*Pseudomonas syringae* pv. *actinidiae* (Psa) is the most destructive pathogen of kiwifruit, and the absence of curative measures makes the management of Psa-induced kiwifruit bacterial canker (KBC) particularly challenging. *Elettaria cardamomum* produces an essential oil (CAR) rich in bioactive compounds with demonstrated potential to act directly against Psa, but its mechanisms of action remain insufficiently explored. Here, we investigated CAR’s mode of action in plants with established mild KBC symptoms, and assessed its potential as a plant elicitor. In the *in planta* assay, CAR application (0.1% *w*/*v*, applied 7 days after inoculation) reduced KBC symptoms, with the strongest effect observed 14 days after treatment (DAT). However, CAR did not significantly affect oxidative stress biomarkers, antioxidant system, pigments and primary metabolism, or the expression of target genes related to systemic acquired resistance or salicylic acid and jasmonic acid pathways. For instance, Psa inoculation significantly upregulated *PR1* (≈5.4-fold) and *PR5* (≈4.3–5.5-fold), irrespective of CAR application. Complementary *in vitro* assays revealed a transient, phase-dependent antimicrobial activity of CAR: although the effect disappeared by 32 h in liquid-phase assay and no inhibition was observed under vapor-phase exposure, a strong reduction in Psa viable cells (79.7%) was observed after 8 h exposure in the liquid phase. This study demonstrates that CAR exerts a direct, albeit transient, antibacterial effect against Psa, conferring curative activity when applied to plants with mild KBC symptoms. Consequently, repeated applications may be required to maintain disease suppression, as CAR does not appear to induce a sustained preventive defense response in the host.

## 1. Introduction

Nearly two decades after the first outbreak of biovar 3 of *Pseudomonas syringae* pv. *actinidiae* (Psa) in Italy, kiwifruit bacterial canker (KBC) remains a problem and is currently the most devastating disease affecting kiwifruit production worldwide [1]. Disease mitigation has relied mainly on improved orchard hygiene, the release of new *Actinidia chinensis* cultivars and the development of novel plant protection formulations [2,3,4]. These formulations must be applied before pathogen infection, either to activate plant defense mechanisms or to support effective colonization by Microbial Biological Control Agents (MBCAs). For this reason, they are mainly used as preventive measures [5,6]. Despite extensive research efforts, complete curative control of Psa *in planta* following symptom onset has yet to be achieved, underscoring the intrinsic difficulty of controlling established infections [4]. Such an approach would require a direct bactericidal activity capable of reducing Psa load in plant tissues to levels compatible with disease recovery, while ensuring minimal environmental impact and no human health risk [3]. Among the most promising substances for curative control, plant essential oils (EOs) have attracted increasing interest due to their high levels of bioactive specialized metabolites. These complex, volatile-rich mixtures extracted from aromatic plants combine biodegradability with economic feasibility. Their intrinsic potency often enables effective control at relatively low doses, making EOs attractive for integration into sustainable phytosanitary programs [7]. However, their poor aqueous solubility and potential phytotoxicity often require emulsification with surfactants, restricting their use to low concentrations, typically ≤1% [8].

EOs consist mainly of terpene hydrocarbons (further divided into monoterpenes and sesquiterpenes) and oxygenated derivatives (including alcohols, simple phenols, polyphenols, aldehydes, ethers and esters) [9]. Despite their complex composition, which typically includes 20 to 60 different molecules, EOs are mostly composed of one to three predominant constituents [9]. Because EOs can act through direct contact or via their volatile fraction, their bioactivity is commonly assessed using *in vitro* assays that help determine the relative contribution of soluble and volatile constituents [10]. EOs have shown potential against viruses, fungi, bacteria and entomofauna [9]. In bacteria, EOs and their major constituents may act through membrane disruption, increased permeability, and interference with virulence-related processes, such as quorum sensing, biofilm formation, secretion of plant cell wall-degrading enzymes and expression of virulence genes [11,12].

Several EOs have been tested against Psa, primarily in laboratory trials. In general, the EOs showing higher anti-Psa potential were those rich in simple phenols (e.g., carvacrol and eugenol), polyphenols (e.g., xanthones and tannins), olefinic compounds (e.g., stilbenes), and aldehydes (e.g., cinnamaldehyde) (reviewed by Santos et al. [4]). Although *in planta* experiments have begun to gain relevance in recent years [13,14], the antimicrobial activity of EOs observed *in vitro* often fails to translate into effective control *in planta* [14]. Moreover, in the few studies evaluating EOs or their main constituents against Psa, applications were predominantly preventive (e.g., from 1 to 3 d) [13,15,16] or at the time of bacterial inoculation [11]. Only a few exceptions have reported EO treatments applied after Psa inoculation (e.g., 24 h) [17], while truly curative approaches targeting plants with established KBC symptoms remain unexplored. Although the curative effects of EOs are often attributed to their direct antimicrobial activity [18], it remains unclear whether plant biochemical and molecular responses also partly contribute to disease mitigation under curative conditions. In a previous study, we assessed the *in vitro* and *in planta* antimicrobial activities of six EOs (anise, cumin, fennel, basil, laurel and cardamom), all applied at a low concentration (0.1%) and selected to avoid the phytotoxic effects observed at higher levels, against Psa. In that work, we showed that *in vitro* efficacy does not necessarily predict *in planta* performance, which depends strongly on the timing of EO application, either before or after infection [14]. Among the tested EOs, cardamom EO (CAR) stood out for its markedly contrasting *in planta* behavior, significantly reducing Psa endophytic population in *A. chinensis* tissues by 70% when applied curatively (14 d after Psa inoculation), but not when applied preventively (14 d before Psa inoculation). This pattern suggests that CAR’s effectiveness may rely primarily on direct antimicrobial activity against Psa rather than on the induction of plant defenses [19], making it a good target for exploring our hypothesis. Importantly, in the aforementioned study, *in planta* assessments were limited to endpoint colony-forming units (CFU) quantification [1], and a comprehensive characterization of CAR’s curative activity, encompassing plant responses and complementary antimicrobial assays, is still missing. Here, we aimed to test the hypothesis that an EO exhibiting strong *in planta* curative activity against a given pathogen may not necessarily trigger plant defense mechanisms that are crucial for enhanced protection against subsequent infections. These defenses typically involve the activation of defense-related genes and modulation of plant redox homeostasis [20,21]. Therefore, the present study aimed to: (i) assess the curative potential of CAR against KBC when applied at the onset of visible symptoms; (ii) characterize host biochemical and molecular responses following CAR application; (iii) evaluate CAR antimicrobial activity through complementary liquid- and vapor-phase *in vitro* assays; and (iv) contribute to the body of knowledge on candidate bioactive molecules with curative efficacy against KBC. The information generated in this study provides insights for optimizing CAR application as a curative strategy against KBC, while improving the understanding of its impact on plant defense responses.

## 2. Results

### 2.1. Volatile Headspace Profile of the Cardamom Essential Oil

**Figure 1:**
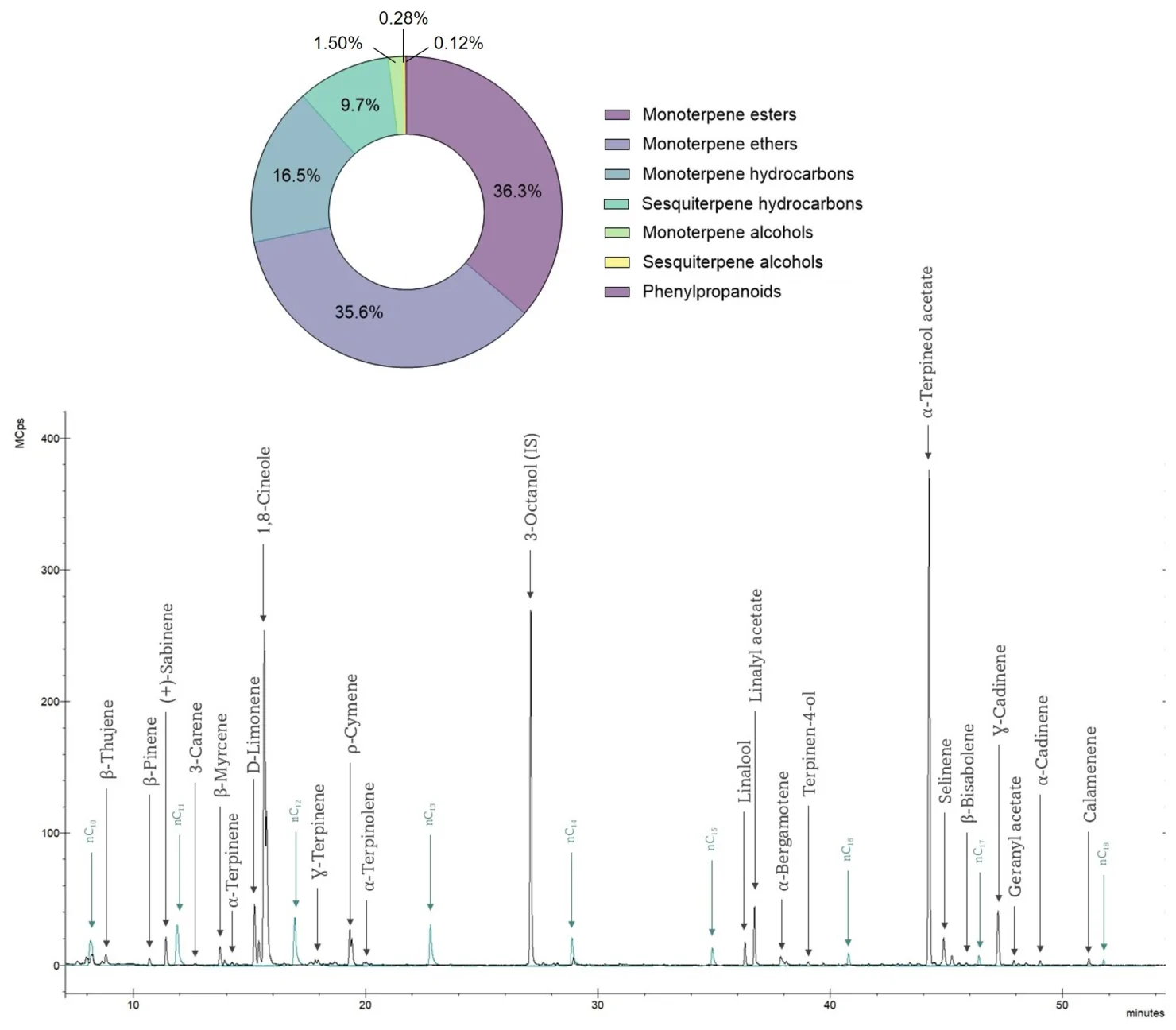
Chemical-class distribution and representative HS-SPME-GC-MS chromatogram of cardamom essential oil. The doughnut chart shows the relative abundance (%) of the main chemical classes detected in the volatile headspace fraction. The representative chromatogram shows the peaks corresponding to the most abundant identified compounds, as well as the internal standard (IS, 3-octanol), indicated by black arrows and labels in the black trace. The n-alkane homologous series used for experimental retention index calculation is shown as a green trace, with alkane peaks indicated by green arrows and labels. The relative abundance of all identified individual constituents is provided in Table 1.

A total of 29 constituents were tentatively identified in the volatile profile of CAR by headspace solid-phase microextraction coupled with gas chromatography–mass spectrometry (HS-SPME-GC-MS; Figure 1, Table 1). The detected profile was dominated by oxygenated monoterpenes, particularly monoterpene esters and monoterpene ethers, which together accounted for approximately 72% of the total chromatographic peak area. Monoterpene hydrocarbons represented 16.5%, followed by sesquiterpene hydrocarbons, which accounted for 9.7% (Figure 1). Minor chemical classes included monoterpene alcohols (1.5%), sesquiterpene alcohols (0.28%), and phenylpropanoids (0.12%).

The major individual constituents were 1,8-cineole (35.6%) and α-terpinyl acetate (32.6%), which together represented more than two thirds of the detected volatile fraction. Other relevant compounds included D-limonene (5.73%), γ-cadinene (4.42%), p-cymene (3.92%), and linalyl acetate (3.35%) (Table 1). Several constituents were detected at low relative abundance, including nerol acetate, eugenol, β-sesquiphellandrene, β-bisabolene, 3-carene, and terpinen-4-ol, each representing less than 0.25% of the total peak area.

### 2.2. In Planta Curative Activity of Cardamom Essential Oil Against KBC

**Figure 2:**
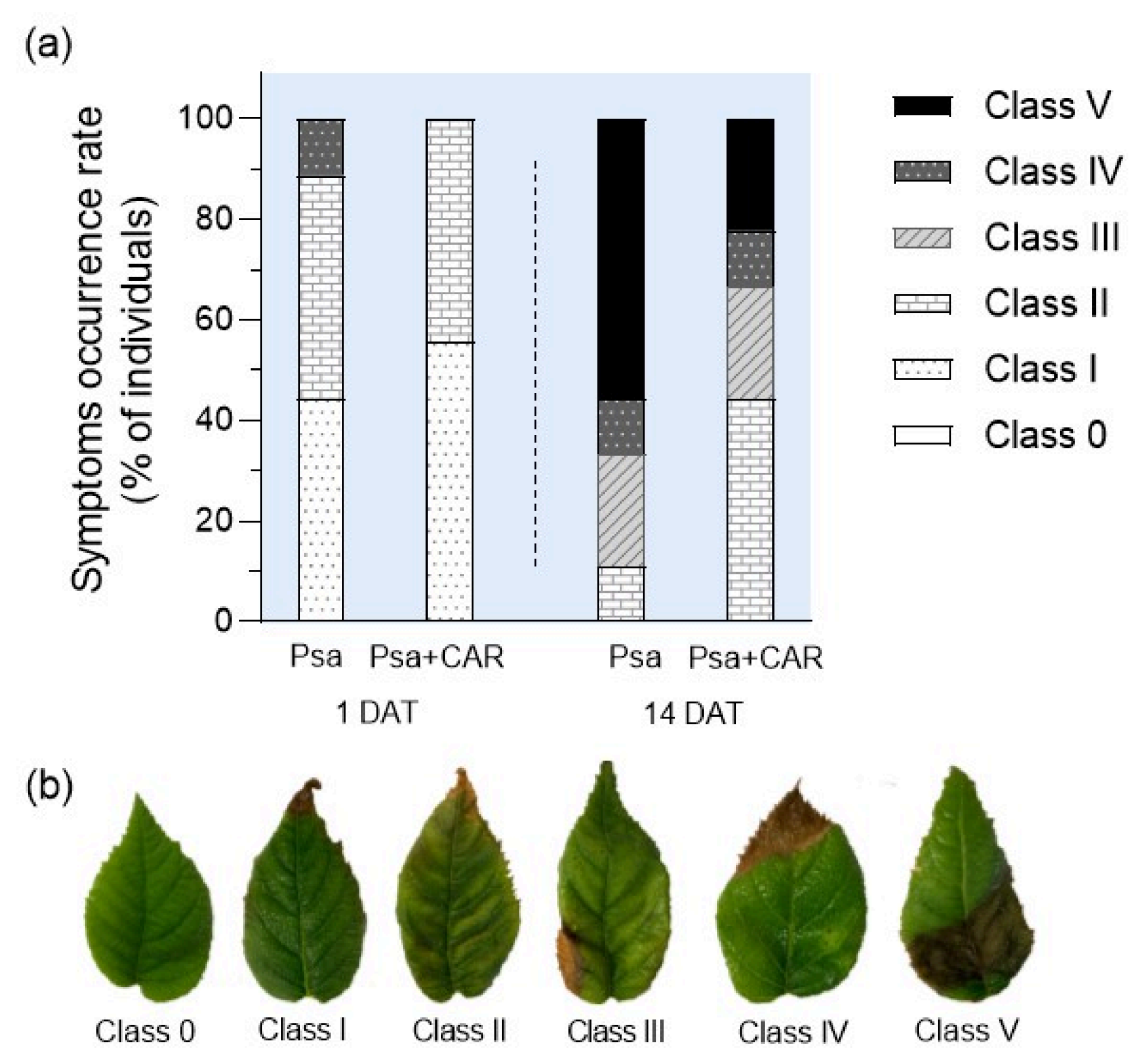
(**a**) Symptom occurrence rate (percentage of individuals showing disease symptoms per severity class) in *Actinidia chinensis* var. *deliciosa* ‘Hayward’ plants inoculated with *Pseudomonas syringae* pv. *actinidiae*, and subsequently treated (‘Psa+CAR’) or non-treated (‘Psa’) with cardamom essential oil. Foliar symptoms were scored taking into account the percentage of leaf area with any signs of damage at 1 and 14 days after treatment (DAT) with the essential oil. Class 0: no lesions; class I: <5%; class II: 5–9%; class III: 10–14%; class IV: 15–19%; class V: >20%. (**b**) Representative leaves from each symptom class.

All plants from both Psa-inoculated groups (‘Psa’ and ‘Psa+CAR’) exhibited symptoms of KBC at 1 DAT (8 dpi), mainly within severity classes I and II (≤9% lesion area; Figure 2). At this time point, in the ‘Psa’ group, 44% of plants were classified as class I, 44% as class II, and the remaining as class IV. By contrast, no plants in the ‘Psa+CAR’ group reached class IV, with all plants classified as class I (56%) or class II (44%). At 14 DAT (21 dpi), KBC symptom progression was observed in both ‘Psa’ and ‘Psa+CAR’ groups, with a general shift toward higher symptom severity classes. However, disease severity remained lower in CAR-treated plants (‘Psa+CAR’ group). Only 22% of ‘Psa+CAR’ plants reached the highest severity class (class V; >20% of lesion area), compared with 56% of plants in the ‘Psa’ group. Moreover, the lowest symptom class observed at this stage (class II) was more prevalent in the ‘Psa+CAR’ (44%) than in the ‘Psa’ (11%) group.

### 2.3. Plant Biochemical and Molecular Analyses

**Figure 3:**
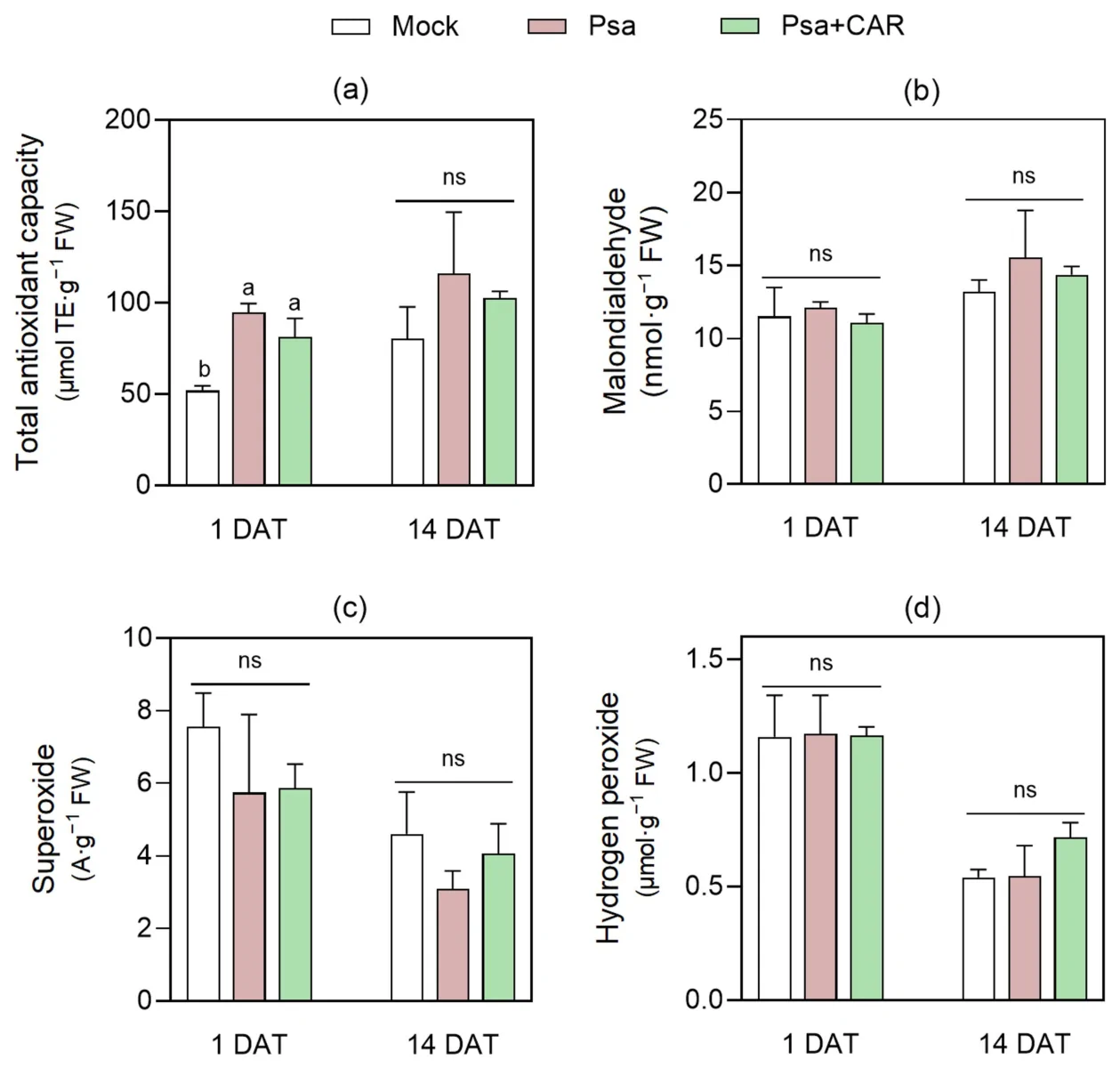
(**a**) Total antioxidant capacity, and concentration of (**b**) malondialdehyde, (**c**) superoxide and (**d**) hydrogen peroxide in *Actinidia chinensis* var. *deliciosa* ‘Hayward’ plants mock inoculated (‘Mock’), or artificially inoculated with *Pseudomonas syringae* pv. *actinidiae*, and subsequently treated (‘Psa+CAR’) or non-treated (‘Psa’) with cardamom essential oil. Results are presented as the mean ± standard error of the mean (*n* = 3; each replicate consisting of a pooled sample of three plants), at 1 and 14 days after treatment (DAT) with the essential oil. Different letters indicate statistically significant differences (ANOVA followed by Tukey’s test, *p* < 0.05). Abbreviations: A = absorbances; FW = fresh weight; ns = non-significant; TE = trolox equivalents.

Overall, the biochemical analyses revealed no significant differences between Psa-inoculated plants treated with CAR (‘Psa+CAR’) and untreated (‘Psa’), for the analyzed parameters at both 1 and 14 DAT (Table 2 and Figure 3). For instance, the total antioxidant capacity at 1 DAT was significantly higher in Psa-inoculated plants compared with mock-inoculated controls (1.8-fold higher; *p* = 0.024), and was also higher in the ‘Psa+CAR’ group relative to ‘Mock’ (1.6-fold higher; *p* = 0.039), with no significant differences between CAR-treated and untreated plants (Figure 3a).

**Figure 4:**
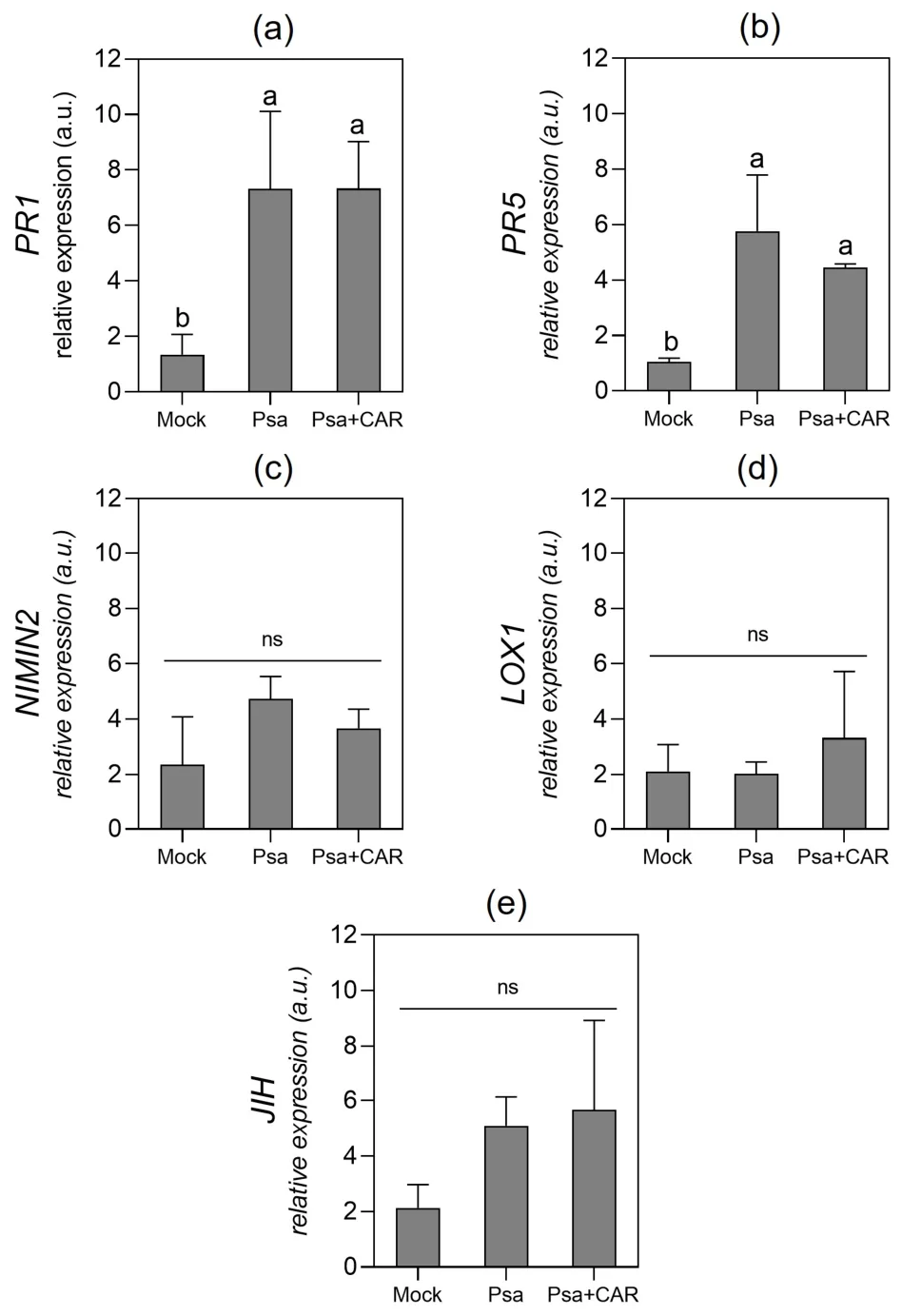
Relative expression of *Pathogenesis-related protein 1* (*PR1*), *Thaumatin-like protein* (*PR5*), *NIM-interacting protein 2* (*NIMIN2*), *Lipoxygenase 1* (*LOX1*) and *Jasmonoyl-isoleucine-12-hydrolase* (*JIH*) (panel (**a**), (**b**), (**c**), (**d**) and (**e**), respectively) in *Actinidia chinensis* var. *deliciosa* ‘Hayward’, mock inoculated (‘Mock’), or artificially inoculated with *Pseudomonas syringae* pv. *actinidiae*, and subsequently treated (‘Psa+CAR’) or non-treated (‘Psa’) with cardamom essential oil. Gene expression was calculated 1 day after CAR application (Figure 6), using *Actin* (*ACT*) and *Protein phosphatase 2A* (*PP2A*) as reference genes, and with the ‘Mock’ treatment as the calibrator. Different letters indicate statistically significant differences according to one-way ANOVA followed by Tukey’s test (*p* < 0.05); ns = non-significant. Owing to biological variability in ΔCq values among ‘Mock’ samples, the mean fold change in this control group does not necessarily equal 1, despite being used as the reference condition.

Regarding oxidative stress biomarkers, no significant differences were found among treatments in any of the time points (Figure 3b–d), with malondialdehyde showing *p* = 0.843 (1 DAT) and *p* = 0.716 (14 DAT), superoxide showing *p* = 0.618 (1 DAT) and *p* = 0.498 (14 DAT), and hydrogen peroxide showing *p* = 0.998 (1 DAT) and *p* = 0.344 (14 DAT). The remaining biochemical parameters also showed no significant differences between treatments at either 1 or 14 DAT, with total phenolics, total flavonoids, anthocyanins, carotenoids, chlorophylls a, chlorophylls b, soluble sugars and insoluble sugars all exhibiting non-significant ANOVA results (Table 2). Despite this, some parameters exhibited ANOVA *p*-values approaching the adopted significance threshold (α = 0.05), with confidence intervals suggesting potential trends that did not reach statistical significance. This was the case for total flavonoids and anthocyanin concentration at 1 DAT, with *p*-values of 0.055 and 0.065, respectively. In particular, mean values indicated higher flavonoid concentration in ‘Psa’ and ‘Psa+CAR’ plants compared with mock-inoculated plants (1.9 and 2.0-fold above ‘Mock’, respectively), although these differences did not reach statistical significance. In the case of anthocyanin concentration, an opposite trend was observed, with lower values registered in the ‘Psa’ and ‘Psa+CAR’ groups (27% and 42% lower, respectively) compared with the plants in the ‘Mock’ group, although this difference was not statistically significant. Gene expression analysis revealed a significant impact of Psa inoculation on *PR1* (*p* = 0.029) and *PR5* (*p* = 0.007) genes, whereas no significant differences were detected for *NIMIN2* (*p* = 0.216), *LOX1* (*p* = 0.806) or *JIH* (*p* = 0.362) (Figure 4). Particularly, *PR1* gene was significantly overexpressed in both the ‘Psa’ (*p* = 0.047; 5.43-fold change) and ‘Psa+CAR’ groups (*p* = 0.041; 5.44-fold change) relative to the ‘Mock’ group. Similarly, *PR5* showed higher expression levels in ‘Psa’ (*p* = 0.009; 5.54-fold change) and ‘Psa+CAR’ plants (*p* = 0.013; 4.28-fold change), when compared with the ‘Mock’ group.

### 2.4. In Vitro Antimicrobial Activity of Cardamom Essential Oil Against Psa

**Figure 5:**
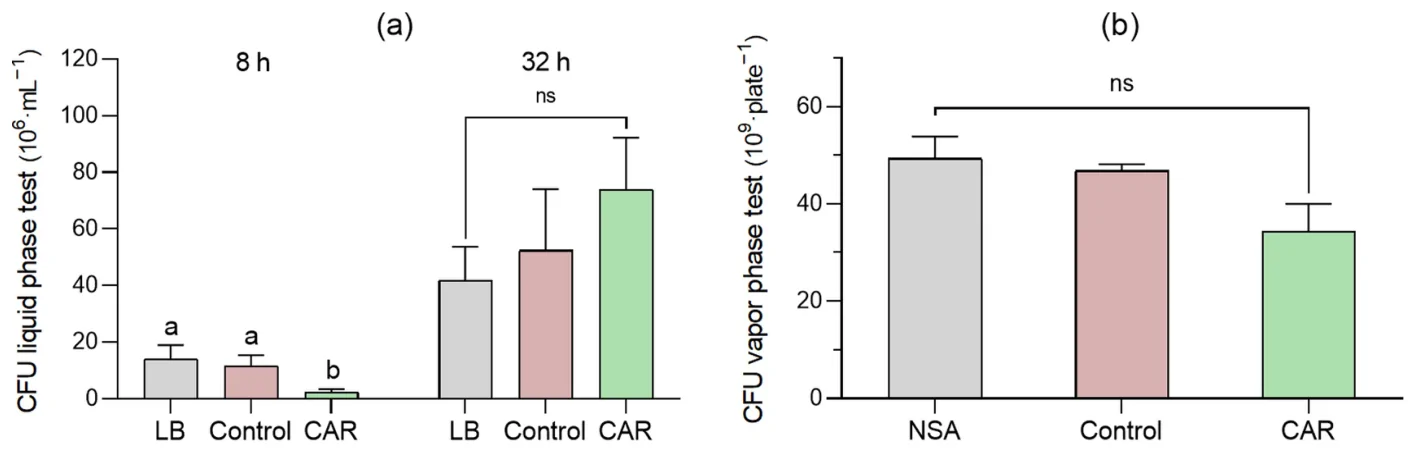
Number of colony-forming units (CFU) of *Pseudomonas syringae* pv. *actinidiae* (Psa) estimated in: (**a**) liquid-phase test at 8 and 32 h of incubation under direct exposure to cardamom essential oil (CAR); and (**b**) vapor-phase test at 48 h of incubation under exposure to the volatile fraction of the CAR in nutrient sucrose agar plates. ‘LB’ and ‘NS’: control without Tween20; ‘Control’: control with 2% Tween20 (*v*/*v*); ‘CAR’: emulsion solution of cardamom essential oil 0.1% (*w*/*v*) 2% Tween20 (*v*/*v*). Error bars represent standard errors of the means. Different letters in graph (**a**) at the 8 h time point indicate significant differences through Tukey’s post hoc test (*p* < 0.05) following one-way ANOVA; ns = non-significant. In (**b**), data were analyzed nonparametrically through the Kruskal–Wallis H test (α = 0.05).

The *in vitro* antimicrobial activity of CAR against Psa varied with the assay type and incubation time (Figure 5). In the liquid-phase assay, CAR treatment (‘CAR’) significantly reduced viable bacterial cell density after 8 h incubation, resulting in a 79.7% decrease relative to ‘Control’ plates (*p* = 0.013; Figure 5a). However, this inhibitory effect was transient, as no significant differences among treatments were detected 32 h after incubation. At both time points, Tween20 surfactant had no impact on bacterial cell density (i.e., ‘LB’ vs. ‘Control’). In the vapor-phase test, the Kruskal–Wallis H test revealed no significant differences in the density of viable cells among treatments (*p* = 0.150), indicating that CAR volatile compounds at the tested concentration did not exert antimicrobial activity against Psa under the experimental conditions (Figure 5b).

**Figure 6:**
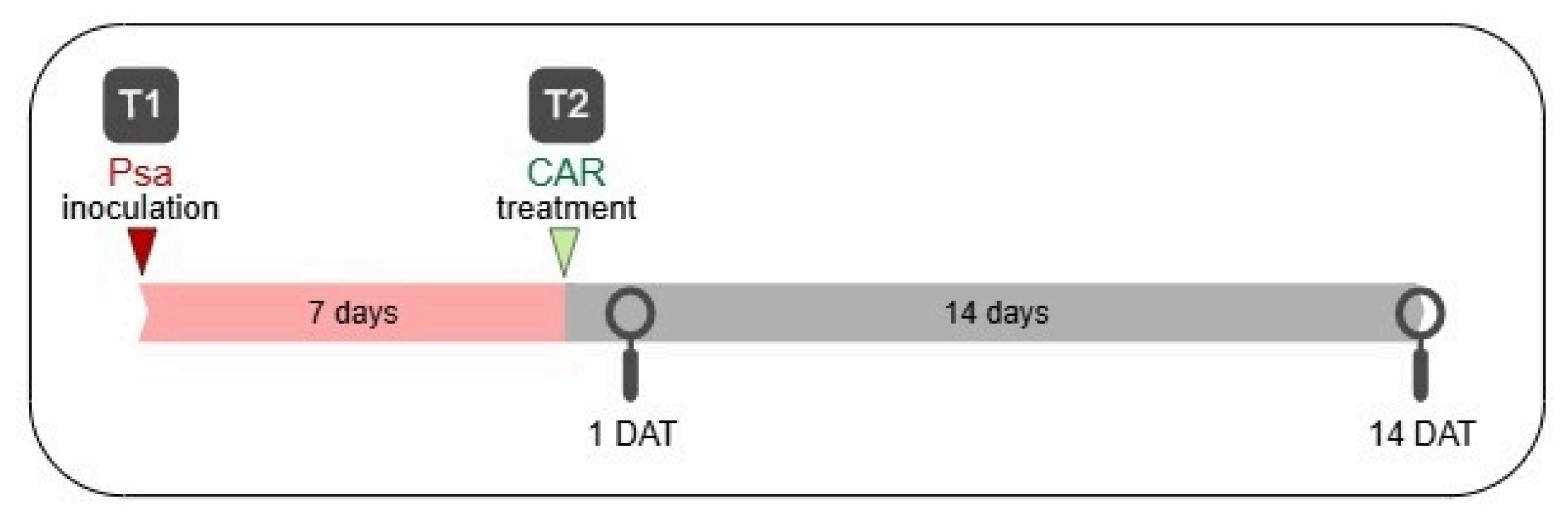
Timeline of the *in planta* experiment, indicating the timing of *Pseudomonas syringae* pv. *actinidiae* (Psa) inoculation (at T1), cardamom essential oil (CAR) application (at T2), and subsequent plant analyses performed with shoot samples collected 1 and 14 days after treatment (DAT) with the CAR.

## 3. Discussion

### 3.1. Volatile Headspace Profile of the Cardamom Essential Oil and Identification of Potential Antimicrobial Compounds Against Psa

In the current study, the volatile headspace profile of CAR was composed primarily of monoterpene esters, monoterpene ethers and monoterpene hydrocarbons, which accounted for 88% of its composition (Figure 1). These chemical groups comprise molecules with antioxidant, antifungal, antiviral, and antibacterial properties, among other biological functions [19,22].

The predominance of oxygenated monoterpenes (e.g., 1,8-cineole, α-terpinyl acetate, linalyl acetate and linalool) and monoterpene hydrocarbons in CARs, is frequently reported [23,24,25,26,27]. Nevertheless, different chemical profiles of CAR have also been documented [14], which may indicate distinct plant chemotypes underlying their origin [28]. For example, the CAR reported in Nunes da Silva et al. [14] was predominantly composed of α-hymachalene (76%), whereas the second and third constituents, namely D-limonene and linalyl acetate, were found at much lower levels (7.2% and 3.7%, respectively). In that case, three constituents accounted for 87% of the CAR, whereas in the current study seven constituents (1,8-cineole > α-terpinyl acetate > D-limonene > γ-cadinene > p-cymene > linalyl acetate > selinene) accounted for 88.3% of the CAR composition. This highlights the importance of EO chemical characterization, as such analyses allow for the identification of candidate molecules for formulating innovative plant protection products tailored to specific control methods (preventive vs. curative) or different pathosystems [9].

In the present study, the CAR was characterized by two clearly predominant constituents, 1,8-cineole and α-terpinyl acetate, embedded within a chemically diverse matrix of monoterpenes and sesquiterpenes. Notably, several of the constituents identified in CAR, including most of those listed above, might also contribute to its bioactivity, namely D-limonene [29], γ-cadinene [30], p-cymene [31], linalyl acetate [32], selinene [33], β-myrcene [34] and (+)-sabinene [35]. This reflects the high complexity of the current CAR, suggesting a wide range of potential interactions among its volatile constituents.

The monoterpene ether class in the present CAR is represented exclusively by 1,8-cineole, which has been shown to disrupt bacterial membrane integrity and interfere with intracellular metabolic pathways [36]. Monoterpene esters, mainly represented in CAR by α-terpinyl acetate and linalyl acetate, have been associated with membrane-targeting antimicrobial effects, with α-terpinyl acetate shown to induce membrane permeabilization and sublethal damage in Gram-negative bacteria [37]. Monoterpene hydrocarbons have generally been associated with membrane perturbation, increasing membrane fluidity and permeability, although their intrinsic antimicrobial activity is typically weak. Several members of this class, including p-cymene, β-myrcene and α-pinene, have been reported to act primarily as synergists, facilitating membrane penetration of oxygenated terpenes and thereby enhancing the overall bioactivity of the EO, rather than exerting direct bactericidal effects [38,39,40]. Together, these findings highlight the chemical complexity of the CAR, in which multiple constituents are present at considerable relative abundances and may act synergistically or antagonistically to determine its biological activity. Although synergistic interactions among monoterpenes are more frequently reported, antagonistic effects have also been described [41]. This compositional diversity may influence both its direct antibacterial effects and its interactions with plant defense mechanisms, although no evidence of the latter was observed under the curative conditions tested in this study, as discussed in the following sections.

### 3.2. In Planta Curative vs. Preventive Activity of Cardamom Essential Oil Against KBC

When applied as a preventive measure, the impact of EOs on plant performance is particularly relevant, since the induction of plant defenses (elicitation) is a major driver of crop protection compared with curative conditions [42]. Conversely, the relevance of elicitation is likely diminished when EOs are applied at the symptomatic stage of KBC, since Psa has already proliferated endophytically and disrupted host homeostasis, thereby favoring disease progression [43,44]. Pathogen control becomes more challenging once the treatment is applied after disease establishment. At the time of the CAR application (7 dpi), the Psa had sufficient time to colonize the plant and to exponentially expand its endophytic population [45]. In addition, this allows Psa to express its arsenal of virulence factors, including ethylene production, further enhancing pathogenicity and increasing plant vulnerability to bacterial colonization [44]. In this study, using *A. chinensis* var. *deliciosa* ‘Hayward’, it was shown that CAR applied at an early symptomatic stage significantly delayed the progression of KBC symptoms, highlighting its strong curative potential (Figure 2a). In general, typical symptoms of KBC can be visually detected after approximately 6 d after Psa infection [46]. The present study was consistent with this, with some plants showing initial symptoms from 5 dpi onward. By 8 dpi (1 DAT), all Psa-inoculated plants exhibited foliar damage (i.e., ‘Psa’ and ‘Psa+CAR’ groups; Figure 2a). The symptoms were primarily leaf necroses (Figure 2b), more extensive than the small, angular spots with yellow halos commonly seen in Psa-infected plants grown in the field. Discrepancies in KBC symptom phenotypes between adult kiwifruit plants and *in vitro* culture plants have been previously reported, presumably linked to rapid plant tissue colonization under *in vitro* conditions and possibly to altered pathogen behavior in such environments [21,47]. This fast leaf damage progression, with plants reaching more than 20% of damaged leaf area within few days, is rarely observed in older plants, whether grown in greenhouse or under field conditions [48,49]. Despite this representing an additional challenge for KBC curative approaches when assessed *in vitro*, the CAR provided significant *in planta* protection against Psa, as reflected in the lower severity of foliar symptoms in the plants treated with this EO. Pathogen loads were not quantified *in planta* in the present work, limiting the interpretation of the link between symptom mitigation and bacterial suppression. Nonetheless, the current results align with our previous study in *A. chinensis* var. *deliciosa* ‘Tomuri’, in which CAR reduced Psa CFUs *in planta*, supporting the interpretation that its direct antibacterial activity is retained within the plant and contributes to the observed symptom reduction [14].

Previous studies have shown that EOs can alleviate KBC symptoms when applied *in planta,* but most were experimental setups in which EOs (or their main constituents) were tested preventively, before Psa inoculation. Examples include foliar application of extracts of *Camellia sinensis* [50], EO of *Thymus vulgaris* [15], *Mentha pulegium*, *Satureja montana* [13], cinnamon bark, oregano and clove bud [17], or EO’s constituents, such as thymol, cinnamaldehyde [51] and forsythoside A (obtained from *Forsythia suspensa*) [11]. For example, Danzi et al. [17] demonstrated that EOs derived from cinnamon bark, oregano, clove bud and thyme (species names not disclosed) provided the greatest protection to kiwifruit plants when applied 24 h prior to Psa inoculation. This timing likely enhanced antimicrobial activity at the plant surface by increasing EO contact at both the outer and inner phyllosphere with the pathogen [17]. Although most EO constituents are highly to moderately volatile, the persistence of their less volatile fractions can leave a residual protective layer [52]. When inoculation occurs shortly after EO application (e.g., 24 h), this residue may directly inhibit the pathogen, leading to an apparent elicitation effect that likely reflects direct antimicrobial action rather than true activation of plant defenses.

EOs may enhance disease resistance indirectly by inducing plant antioxidant defenses [21] or directly through their intrinsic antioxidant capacity [53]. Antioxidant activity has been reported for major CAR constituents, including 1,8-cineole, α-terpinyl acetate, linalyl acetate [54], α-pinene [55] and linalool [39], among others. However, despite the potential contribution of CAR to antioxidant-related responses, its overall impact may be limited in inherently antioxidant-rich plant species such as *A. chinensis* [56]. Consistently, increased total antioxidant capacity was detected only when comparing mock and Psa-inoculated plants, and no differences were found between CAR-treated and untreated-plants (Figure 3a). This increase is likely attributed to infection-induced flavonoid accumulation, as levels were higher in Psa-infected plants at 1 DAT (Table 2). Enhanced flavonoid biosynthesis in response to Psa infection has been previously reported and is regarded as part of a broader antioxidant response aimed at counteracting the early ROS burst associated with defense signaling and direct combat against the pathogen [20,57]. Indeed, Psa infection rapidly induced ROS (e.g., O_2_^•−^ and H_2_O_2_) accumulation, which may persist for extended periods and lead to oxidative stress [21,57]. In the present study, however, none of the evaluated oxidative stress biomarkers were significantly affected at the assessed time points (Figure 3b–d). This may reflect a partial ability of ‘Hayward’ plants to mitigate oxidative damage despite symptom progression, but may also be influenced by experimental factors such as inoculum concentration or strain virulence.

Overall, the biochemical analyses indicated no meaningful effect of the CAR on the evaluated plant traits, possibly reflecting the transient nature of EO-induced responses [42]. To address this hypothesis, two time points were selected: an early sampling point aimed at capturing rapid and potentially transient responses following CAR application (i.e., 8 dpi, 1 DAT), and a later time point intended to assess longer-lasting or cumulative effects, as well as responses linked to KBC progression and symptom aggravation (i.e., 21 dpi, 14 DAT). The latter time point was expected to capture potential differences in the accumulation of specialized metabolites involved in defense against Psa (e.g., phenolic compounds), as well as metabolic adjustments related to oxidative stress or primary metabolism (e.g., carbohydrates and photosynthetic pigments) that could reflect treatment-related effects on plant fitness [58]. However, none of these responses differed significantly between untreated and CAR-treated plants. It should be noted, however, that the absence of differences at these specific time points does not exclude the occurrence of defense-related responses outside the present sampling window or involving pathways not assessed here. Some variables (total flavonoids and anthocyanins; Table 2) nevertheless showed *p*-values close to the significance threshold, pointing to potential trends that should be interpreted cautiously, as sample size may influence the ability to detect subtle biochemical adjustments. Taken together, however, the available evidence suggests that the reduction in KBC symptoms severity observed in CAR-treated plants is likely attributable primarily to the direct interaction between CAR and the pathogen, rather than to CAR-mediated modulation of plant physiology.

Gene expression analysis was performed at 1 DAT to capture fast and potentially transient defense responses triggered either by the CAR treatment or by the ongoing Psa infection. This assessment was relevant to determine whether the CAR could enhance early plant defenses or, at least, avoid compromising them once the infection had progressed to a symptomatic stage. Among the analyzed genes, *PR1* and *PR5*, two classical markers of SA-dependent SAR [59], were significantly overexpressed in Psa-inoculated plants, independently of CAR treatment. This indicates that such transcriptional activation was primarily driven by the pathogen itself, as mock-inoculated plants displayed markedly lower expression levels. Moreover, the absence of differential expression between the ‘Psa’ and ‘Psa+CAR’ groups suggests that CAR did not further stimulate SAR-related pathways at this early stage after its application, which is consistent with the curative nature of CAR [14] and with the infection already well established at 7 dpi. The inclusion of *NIMIN2*, a regulator of SA accumulation and Nonexpressor of Pathogenesis-Related genes 1 (NPR1)-associated signaling, aimed to verify whether CAR could modulate upstream components of the SA pathway [59]. However, no significant differences were detected among treatments, reinforcing the idea that CAR treatment did not interfere with SA biosynthesis or signaling. Likewise, *LOX1* and *JIH*, two JA-related genes, were included to evaluate potential shifts in the SA–JA signaling interplay [60], but showed no significant transcriptional changes. This stability suggests that CAR neither enhanced nor disrupted the JA-related pathways, which is relevant given the importance of maintaining a balanced SA–JA crosstalk for effective defense against hemibiotrophic pathogens such as Psa. Although SA- and JA-related pathways are known to play key roles in the management of KBC, particularly through SAR activation and the SA–JA trade-off, the CAR did not appear to modulate these hormonal routes, at least within our sampling window. This contrasts with preventive EO applications reported in other studies, where modulation of hormonal signaling is a major component of the protective effect [13]. In the present curative context, however, the transcriptional profile of SAR- and SA/JA-related markers indicates that CAR’s contribution to symptom mitigation was not primarily mediated by early hormonal reprogramming, but is more plausibly associated with a direct interaction with the pathogen.

Despite the transient antibacterial activity of CAR observed *in vitro*, where inhibition of *Psa* was no longer detected after 32 h of exposure (Figure 5), CAR-treated plants showed reduced KBC symptom severity. This outcome is likely influenced by the timing and physiological context of CAR application. At 7 dpi, *Psa* is still actively proliferating within the apoplast, and a temporary impairment of bacterial fitness during the early stages after treatment may have been sufficient to limit subsequent tissue colonization [61]. This mechanistic interpretation is consistent with previous reports showing that monoterpenes such as 1,8-cineole can markedly suppress quorum-sensing-regulated virulence traits without causing irreversible loss of microbial viability [62]. Such sub-lethal interference with bacterial physiology, as demonstrated in earlier studies, can reduce colonization efficiency and the expression of key pathogenicity factors, even when cell viability is partially preserved [38]. These early physiological constraints provide a plausible explanation for the reduced symptom progression observed *in planta* despite the transient antimicrobial activity detected *in vitro*. Furthermore, the behavior and persistence of CAR constituents *in planta* are likely to differ substantially from those observed *in vitro*, as interactions with plant tissues and the apoplastic environment may alter their bioavailability and local distribution [14]. Collectively, these observations suggest that the curative effect of CAR may result primarily from an early, direct antibacterial action that temporarily restricts *Psa* proliferation, rather than from a prolonged antimicrobial effect or the activation of plant defense responses. Future studies should investigate whether CAR application at symptom onset effectively limits bacterial spread into newly infected plant tissues.

### 3.3. In Vitro Antimicrobial Activity of Cardamom Essential Oil Against Psa

To better understand the mechanisms of action of CAR, the biochemical and molecular analyses performed *in planta* were complemented with liquid- and vapor-phase *in vitro* assays. In the first one, a significant decrease in Psa cell viability was observed after 8 h of exposure to CAR (Figure 5a). However, this inhibitory effect was transient, as the differences were no longer evident at the end of the assay (Figure 5a). Such transient effects may have practical implications, since a similar effect could occur *in planta*, potentially requiring repeated EO applications without causing phytotoxicity [12].

The short duration of antimicrobial activity has been recognized as one of the main limitations of EOs [63], and may account for the time-dependent loss of inhibitory action against Psa observed in our *in vitro* assay (Figure 5a). This limited persistence is largely attributed to the high volatility of EOs’ constituents, which is strongly and negatively correlated with their molecular weight and positively correlated with exposure temperature [64]. The transient antimicrobial activity of CAR may also be explained by the mode of action of its major constituents. α-Terpinyl acetate (also referred to as α-terpineol acetate), a predominant component of CAR [28], has been shown to disrupt both the outer and the cytoplasmic membranes of bacteria. In *Escherichia coli*, exposure to α-terpinyl acetate at 0.2 µL/mL resulted in approximately four log_10_ cycles of population decline after 5 h. However, its lytic activity appears limited, primarily inducing membrane damage that increases bacterial permeability rather than causing irreversible cell disruption. Bacterial cells may recover from such injuries caused by α-terpinyl acetate, potentially through peptidoglycan synthesis for membrane integrity recovery [65]. At the same dose used for α-terpinyl acetate, 1,8-cineole (0.2 µL/mL) was also found to produce a markedly weaker antimicrobial response, acting predominantly as a transient permeabilizer rather than a true microbicidal agent [37]. Aridoĝan et al. [65] pointed out the inefficacy of 1,8-cineole against Gram-negative bacteria, including *P. aeruginosa*, but reported its effective antimicrobial activity against the Gram-positive *Staphylococcus aureus*. A similar trend was observed for an EO rich in 1,8-cineole (>77%), extracted from the leaves of *Eucalyptus alba* L., which showed moderate activity against *P. aeruginosa* [66]. Moreover, Mutlu-Ingok and Karbancioglu-Guler [25] reported that a CAR whose levels of α-terpinyl acetate (43%) and 1,8-cineole (29%) were comparable to those found in the CAR used in the present study exhibited antimicrobial activity against *Campylobacter* spp., affecting the bacterial cellular homeostasis without showing remarkable lethality. For example, exposure to 1,8-cineole could severely affect both *E. coli* and *S. aureus*, by rupturing both the cell wall and membrane, and causing severe damage to the nucleoplasm [67].

Interestingly, the efficacy of EOs applications for controlling KBC *in planta* appears to be closely linked to the chemical composition of the oils. In this context, the relative abundance of major constituents may influence whether EO activity is primarily associated with direct antibacterial effects rather than host-mediated responses. Regarding Psa, a fennel (*Foeniculum vulgare*) EO rich in 1,8-cineole (69%) and containing a substantial proportion of the sesquiterpene β-himachalene (17%) was shown to significantly reduce Psa CFUs in *A. chinensis* var. *deliciosa* ‘Tomuri’ plants in *in vitro* culture [14]. Importantly, this EO conferred protection only when applied curatively, at 14 dpi, but not when applied preventively [14]. This observation supports the hypothesis that 1,8-cineole primarily exerts a direct antibacterial effect against Psa during symptom progression. Our results are consistent with this interpretation and suggest that 1,8-cineole may partially account for the reduction in KBC symptom severity observed in CAR-treated plants. Nevertheless, the overall *in planta* efficacy of CAR is unlikely to rely on a single constituent alone. Instead, it is more likely driven by the combined and potentially synergistic action of multiple EO constituents, whose relative proportions may critically modulate antimicrobial performance under biologically complex conditions.

Therefore, a detailed understanding of EOs’ composition and modes of action is pivotal for designing novel formulations of plant protection products and optimizing phytosanitary programs. Given that EOs generally exhibit lower antimicrobial activity than synthetic compounds [63], their biological efficacy largely relies on complementary modes of action and synergistic interactions among their constituents [17]. To this end, the vapor-phase assay was used to assess the antimicrobial activity of the volatile fraction of the CAR. This fraction is, in general, the most bioactive in EOs and is also the largest, typically accounting for 90–95% of their composition [10]. Nevertheless, the vapor phase test revealed that the volatile fraction of the CAR—applied at a 0.1% (*w*/*v*) concentration to the filter paper disks—had no significant bactericidal activity against Psa (Figure 5b).

Finally, although CAR showed only a short-lived antibacterial effect under liquid-phase conditions, its application *in planta* still resulted in a clear reduction in the onset and progression of KBC symptoms. This suggests that even a brief reduction in bacterial viability early in the infection is enough to slow disease development. It is also possible that CAR behaves differently in plant tissues, thus persisting longer or interacting with host components in ways that enhance its suppressive effect. These factors together may explain why CAR provided more durable protection *in planta* than would be expected from its short-lived antibacterial effect *in vitro*.

The transient antibacterial activity of CAR observed under liquid-phase conditions, followed by bacterial regrowth after 32 h, likely reflects a combination of physicochemical and bacterial adaptive processes. EOs’ composition changes over time due to differential volatility and oxidative degradation of terpenes and terpenoid constituents [68], progressively reducing the concentration of bioactive molecules below inhibitory levels. In parallel, surviving bacterial cells may resume growth through adaptive stress responses, including membrane remodeling and reinforcement of the peptidoglycan layer, as previously reported for monoterpene constituents with similar modes of action [37,38]. The absence of activity under vapor-phase conditions may further reflect the predominance of highly volatile but less bioactive constituents in the vapor fraction, while less volatile compounds that contribute substantially to antimicrobial activity remain largely in the liquid phase. Together, these factors highlight the importance of both oil stability and mode of exposure in determining antimicrobial efficacy.

Whether the transient inhibition observed *in vitro* primarily reflects sub-lethal membrane perturbation followed by bacterial recovery, or instead a more complex interplay between CAR depletion and bacterial stress-adaptation responses, as suggested in prior studies, remains unresolved and warrants targeted mechanistic investigation.

## 4. Materials and Methods

### 4.1. Media and Chemicals

Cardamom essential oil (CAR) was obtained from *Elettaria cardamomum* seeds (H. Reynaud & Fils, Montbrun-les-Bains, France; batch SDHE23/49/0028; plant origin: India) and Tween20 (Sigma-Aldrich, St. Louis, MO, USA) was used as an emulsifier to prepare CAR emulsions (see Section 4.2).

Murashige & Skoog (MS) medium (Duchefa Biochemie, Haarlem, Netherlands) supplemented with vitamins, sucrose and phytoagar (Duchefa Biochemie) was used for *in vitro* maintenance of *A. chinensis* plantlets (see Section 4.3.1). Nutrient agar (Oxoid, product reference CM0003B, UK) was used to prepare Nutrient Sucrose Agar (NSA) for routine maintenance of Psa cultures (see Section 4.3.2). Luria–Bertani (LB) broth (1% tryptone, 0.5% yeast extract, 1% NaCl) was used for bacterial growth prior to inoculum preparation and in the liquid-phase assay exposure (see Section 4.3.2 and Section 4.4). Ringer’s solution (at ¼ strength; 1.8 g·L^−1^ NaCl, 0.043 g·L^−1^ CaCl_2_ and 0.093 g·L^−1^ KCl; pH 7.4) was used to prepare bacterial suspensions (see Section 4.3.2).

### 4.2. Volatile Headspace Profiling of Cardamom Essential Oil by SPME-GC-MS and Emulsion Preparation

For volatile profiling, the CAR container was opened fresh and subsequently stored hermetically sealed at −18 °C in its original container until subsequent use. For the volatile headspace profile of CAR, the oil was analyzed by HS-SPME-GC-MS. CAR was diluted 1:500 in GC-grade ethanol, and 20 µL of 3-octanol solution (50 mg·L^−1^) were added as an internal standard. Ten milliliters of the diluted sample were transferred to a sealed headspace vial and equilibrated at 40 °C for 2 min. Volatile compounds were then extracted by exposing a 50/30 µm divinylbenzene/carboxen/polydimethylsiloxane (DVB/CAR/PDMS) SPME fiber (Supelco, Bellefonte, PA, USA) to the sample headspace for 40 min at 40 °C. After extraction, the SPME fiber was introduced into the GC injector and thermally desorbed at 220 °C for 10 min. Analyses were performed using an EVOQ Triple Quadrupole mass spectrometer coupled to a 456-GC system (Bruker, Karlsruhe, Germany). Helium was used as the carrier gas at a constant flow rate of 1.0 mL·min^−1^, and the injector temperature was set to 250 °C. The oven temperature program was as follows: initial temperature of 40 °C held for 1 min, increased to 220 °C at 2 °C·min^−1^, and held at 220 °C for 5 min. Mass spectra were acquired in the range *m/z* 33–350. Detected compounds were identified using Bruker Daltonics MS Workstation v8.2.1 by comparison of their mass spectra with the NIST Tandem Mass Spectral Library v2.3 and the InChI Library v1.05. Compound identification was further supported by comparison of experimental linear retention indices, calculated using an n-alkane homologous series analyzed under the same chromatographic conditions, with values reported in the literature, and relative peak areas were calculated by peak area normalization and expressed as percentage of the total chromatographic area. A 500 mL CAR emulsion (0.1% *w*/*v*, a concentration previously selected to avoid phytotoxicity in kiwifruit tissues [14]) was prepared by adding 547 µL of CAR (density 914 g·L^−1^) to sterile deionized water containing Tween20 (2% *v*/*v*). The mixture was homogenized by vigorous manual shaking for 1 min. The emulsion was prepared immediately before use and homogenized again immediately before each plant submersion, and was not stored.

### 4.3. In Planta Assessment of CAR’s Curative Potential

#### 4.3.1. Plant Material and Growth Conditions

Micropropagated plants of *A. chinensis* var. *deliciosa* ‘Hayward’ (Cultigar, S.L., A Coruña, Spain), of 4–6 cm in height (excluding *calli*) with 5–7 leaves, were maintained on MS medium supplemented with vitamins, 30 g·L^−1^ sucrose and 7 g·L^−1^ phytoagar. Plantlets were grown in a Fitoclima 12.000 PLH climate chamber (Aralab, Rio de Mouro, Portugal) at 23/20 °C (light/dark), with a 16 h photoperiod and 200 μmol·m^−2^·s^−1^ photosynthetic photon flux. At the start of the experiment, the *in vitro* plants measured 4–6 cm in height and had 5–7 leaves.

#### 4.3.2. Bacterial Growth Conditions and Inoculum Preparation

The Psa strain 7286 (Collection Française de Bactéries Phytopathogènes) was maintained on NSA medium, as described elsewhere [14,69]. For inoculum preparation, a single colony was transferred to 30 mL of LB broth, and incubated in the dark for 16 h at 26 °C, an overnight growth period commonly used for Psa inoculum [5,14,70], with 50 rpm agitation in horizontally placed 50 mL tubes. Cultures were then centrifuged (5 min; 1800× *g*), and the pellet resuspended in ¼-strength Ringer’s solution. To prepare inoculum, the absorbance (OD_600_) of bacterial suspension was adjusted to 0.10, corresponding to 1–2 × 10^7^ CFU·mL^−1^.

#### 4.3.3. Plant Inoculation, CAR Treatment and Sampling

Plants were inoculated by submerging shoots in the Psa suspension for 15 s with gentle shaking, then air-dried for 15 s and returned to their flasks (time point 1; Figure 6). CAR treatment was applied 7 days post-inoculation (dpi) (time point 2; Figure 6), corresponding to the onset of visible symptoms as reported in the EPPO guidelines for Psa [46]. This timing ensured a curative application, with a mild infection already established. CAR application to the plants (‘Psa+CAR’ group) followed the same procedure, where shoots were submerged for 15 s with gentle shaking in freshly homogenized CAR emulsion, using 60 mL sub-batches that were renewed after every three plantlets to ensure consistent exposure. Negative control plants were mock-inoculated (‘Mock’) with ¼-strength Ringer’s solution at the first time point (T1) and treated with the 2% Tween20^®^ solution (without CAR) at T2. Positive controls (‘Psa’) were inoculated with Psa at T1 and treated with the 2% Tween20^®^ solution (without CAR) at T2. After T2, plants were cultivated for 14 d. Shoots were sampled 1 and 14 days after treatment (DAT) with CAR. Three biological replicates (*n* = 3) were collected per time point, each consisting of pools of three plants (total of 72 plants: 4 treatments × 2 sampling time points × 3 plants per pool × 3 replicates). Samples were flash-frozen in liquid nitrogen and maintained at −80 °C.

#### 4.3.4. KBC Symptoms Occurrence Rate

KBC foliar symptoms were scored based on the percentage of necrotic tissue relative to total leaf area, using the following scale: class 0: no lesions; class I: <5% necrosis; class II: 5–9%; class III: 10–14%; class IV: 15–19%; class V: >20%. Symptoms were evaluated in nine plants (*n* = 9) per treatment, at 1 and 14 DAT.

#### 4.3.5. Plant Biochemical and Molecular Analyses

Biochemical and molecular parameters were assessed in shoot samples macerated in liquid nitrogen. Three biological replicates were prepared (*n* = 3), each consisting of a pooled sample of three plants. Aliquots were prepared and stored at −80 °C until analysis. The biochemical analyses included a total of 12 parameters performed 1 and 14 DAT, whereas the molecular analyses were only conducted on samples collected at 1 DAT, focusing on the expression of five defense-related genes.

##### Oxidative Stress Biomarkers

Lipid peroxidation (malondialdehyde concentration)

Lipid peroxidation was estimated by quantifying malondialdehyde (MDA) following Heath and Packer [71] with adaptations. Sample aliquots of 100 mg of shoot fresh weight (FW) were added to 1 mL of a 0.1% (*w*/*v*) trichloroacetic acid (TCA) solution and homogenized in a TGrinder H24R Tissue Homogenizer (Tiangen Biotech, Beijing, China) at 3800 rpm and linear speed of 6.25 m·s^−1^ during 30 s at 4 °C, with a 10 s pause (three cycles). The homogenates were centrifuged at 12,000× *g* for 15 min at 4 °C. Then, 250 μL of the supernatant was mixed with 1 mL of 0.5% (*w*/*v*) thiobarbituric acid in 20% TCA (*w*/*v*). After incubation at 95 °C for 30 min, samples were cooled on ice for 10 min and centrifuged at 10,000× *g* for 15 min. Absorbance was measured in a Multiskan SkyHigh Microplate Spectrophotometer (ThermoFisher Scientific, Vantaa, Finland). All absorbance readings listed below were obtained using this device. MDA concentration (nmol·g^−1^ FW) was calculated using Equation (1):

(1) $$MDA=\frac{\left(A_{532}-A_{600}\right)\;\times \;V\;\times \;1000}{\varepsilon \times m}$$

where A_532_ and A_600_ are the absorbances at 532 nm and 600 nm, respectively, *V* is the extraction volume (mL), ε is the molar extinction coefficient (ε = 155 mM^−1^·cm^−1^) and *m* is the shoot biomass (g FW).

Hydrogen peroxide quantification

H_2_O_2_ was quantified using the method described in Alexieva et al. [72]. Shoot samples (100 mg FW) were homogenized in 1.5 mL 0.1% (*w*/*v*) TCA using the same homogenizer settings described for lipid peroxidation. The homogenates were centrifuged at 12,000× *g* for 15 min at 4 °C, and 500 μL of the supernatants were transferred to new microcentrifuge tubes, and 500 μL of 0.1 M phosphate buffer (pH = 7.0) and 1 mL of 1 M potassium iodide (KI) were added. Tubes were maintained in the dark for 1 h with inversion every 10 min, and absorbance was read at 390 nm. H_2_O_2_ concentration was calculated from a standard curve and expressed as μmol·g^−1^ FW.

Superoxide radical ion quantification

The concentration of O^2•–^ was quantified from 100 mg FW of shoot tissue homogenized in 1.5 mL of 0.01 M phosphate buffer (pH 7.8), 0.05% (*v*/*v*) Nitro Blue Tetrazolium (NBT) diluted in 100 μL of dimethyl sulfoxide and 10 mM sodium azide, using the same homogenizer settings as in lipid peroxidation. Samples were incubated for 1 h at room temperature (RT), with inversion every 10 min, and centrifuged at 13,000× *g* for 2 min at 4 °C. A 1.5 mL aliquot of the supernatant was incubated at 85 °C for 15 min, then cooled on ice for 10 min. Samples were prepared in triplicate and absorbance of 150 μL of the supernatant was read at 580 nm, using the extraction buffer as the blank. Results were expressed as absorbance units per gram of FW (A·g^−1^ FW).

##### Total Antioxidant Capacity and Non-Enzymatic Component of the Plant Antioxidant System

Total antioxidant capacity

The total antioxidant capacity was assessed using the 2,2′-azino-bis(3-ethylbenzothiazoline-6-sulfonic acid) (ABTS) assay, following the method of Gonçalves et al. [73], with adaptations. Shoot samples (150 mg FW) were homogenized in 1.5 mL 80% (*v*/*v*) methanol using the same homogenizer settings as in lipid peroxidation. Extracts were centrifuged at 13,000× *g* for 15 min at 4 °C, and the supernatants were kept on ice. An ABTS stock solution was prepared by mixing a 7 mM solution of its diammonium salt with a 2.44 mM potassium persulfate solution in a 1:1 ratio in aluminum foil-wrapped tubes, and mixed for 16 h with a magnetic stirrer (500 rpm) in the dark. The resulting ABTS^•+^ solution was filtered (0.45 μm) and diluted with 80% methanol to an absorbance of 0.70 at 734 nm. For total antioxidant capacity determination, 180 μL of ABTS^•+^ working solution was added to 20 μL of extract in a 96-well microplate. Methanol served as the blank, and a Trolox standard curve was used to calculate antioxidant capacity as the percentage reduction in absorbance relative to the blank. Mixtures were incubated for 5 min at 30 °C in the dark, and the absorbance was recorded at 734 nm. ABTS^•+^ scavenging capacity (%) was calculated using Equation (2):

(2) $$ABTS\;scavenging\;\left(A_{Blank}-\;A_{Sample}\right)\times \;\frac{100}{A_{Blank}}$$

where *A_Blank_* is the absorbance of the blank and *A_Sample_* is the absorbance at the sample (or Trolox standard). Total antioxidant capacity was expressed as Trolox Equivalents (TE). Final values were calculated from the Trolox standard curve and expressed as μmol TE·g^−1^ FW.

Total phenolics content

Total phenolics were quantified using the Folin–Ciocalteu colorimetric method [74]. In microtubes, 200 μL deionized H_2_O, 50 μL methanolic extracts of the shoot samples, and 50 μL of 20% Folin–Ciocalteu reagent were mixed for 5 s. For the blank and calibration curve, the sample extract was replaced with 80% methanol and gallic acid standards (prepared in 80% methanol), respectively. After 6 min incubation at RT in the dark, 500 μL of 7% (*w*/*v*) Na_2_CO_3_ and 400 μL of deionized H_2_O were added. After incubation for 90 min in the dark at RT, with inversion every 10 min, absorbance was measured at 760 nm using a microplate reader. Total phenolics were calculated from the gallic acid standard curve to be expressed as Gallic Acid Equivalents (μg GAE·g^−1^ FW).

Total flavonoids

Total flavonoids were quantified using an adaptation of the aluminum chloride (AlCl_3_) method [75]. In 15 mL tubes, 2 mL ultrapure water and 150 μL of 5% (*w*/*v*) sodium nitrite (NaNO_3_) were added to 100 μL methanolic extracts of the shoot samples, and incubated for 5 min at RT. Then, 150 μL AlCl_3_ was added and the samples were incubated for 6 min. Then, 1 mL of 1 M sodium hydroxide (NaOH) and 1.2 mL ultrapure water were added and mixed. Absorbance was recorded at 510 nm in a microplate reader. Total flavonoids (*C_f_*) were calculated from a catechin standard curve using Equation (3):

(3) $$C_{f}=\;\frac{c\;\times V}{m}$$

where *c* is the catechin concentration (mg·mL^−1^) obtained from the standard curve, *V* is the extraction volume (mL), and *m* is the shoot biomass (g FW). Results are presented as Catechin Equivalents (mg CE·g^−1^ FW).

Anthocyanins

Anthocyanins were quantified using an adaptation of Taghavi et al. [76]. Leaf samples (50 mg FW) were homogenized in 300 μL methanol with 1% (*v*/*v*) hydrochloric acid (HCl) using the same homogenizer settings as in lipid peroxidation. Extracts were kept at −20 °C for 16 h to complete pigment extraction. Then, 200 μL ultrapure water and 500 μL chloroform were added and mixed for 5 s. Samples were centrifuged at 18,000× *g* for 5 min at 4 °C, and supernatant was transferred to clean tubes. Absorbance (530 and 657 nm) was measured using 100 μL aliquots. Anthocyanin concentration (*C_a_*) was calculated using Equation (4):

(4) $$C_{a}=\;\frac{\left(A_{530}-\left(0.3\;\times \;A_{657}\right)\right)\;\times \;V}{m}$$

where *A*_530_ and *A*_657_ are the absorbances at the respective wavelengths, *V* is the extraction volume (mL), and *m* is the shoot biomass (g FW). Results are presented as absorbance units per gram of FW (A·g^−1^ FW).

##### Chlorophylls a and b and Carotenoids

Chlorophylls a and b and carotenoids were quantified through a procedure adapted from Lichtenthaler and Wellburn [77], using 80% (*v*/*v*) methanol as the extractant. Aliquots containing 150 μL of the methanolic extracts of the plant samples were transferred to a microplate, and absorbance was recorded at 470, 537, 647 and 663 nm. Concentrations of chlorophylls a [Cha], chlorophylls b [Chb], and carotenoids [Car], in the extracts (μmol·mL^−1^) were calculated using Equations (5), (6) and (8), respectively. Equation (7) was used solely as a correction factor for anthocyanin [Ant] interference at 470 nm, incorporated into Equation (9).

(5) $$\left[Cha]=\left(0.01373\times A_{663}\right)-\left(0.000897\times A_{537}\right)-(0.003046\times A_{647}\right)$$

(6) $$\left[Chb]=\left(0.02405\times A_{647}\right)-\left(0.004305\times A_{537}\right)-(0.005507\times A_{663}\right)$$

(7) $$\left[Ant\right]=\left(0.08173\times A_{537}\right)-\left(0.00697\times A_{647}\right)-(0.00228\times A_{663})$$

(8) $$\left[Car\right]=\frac{\left(A_{470}-\left(17.1\times \left(\left[Cha\right]+\left[Chb\right]\right)-9.479\times \left[Ant\right]\right)\right)}{119.26}$$

where *A*_470_, *A*_537_, *A*_647_ and *A*_663_ are the absorbances at the respective wavelengths. Pigment concentrations were converted to μg·mL^−1^ using the molecular masses of 893.5 g·mol^−1^, 907.5 g·mol^−1^ and 550 g·mol^−1^ for chlorophylls a, chlorophylls b and carotenoids, respectively. For each pigment, Equation (9) was used:

(9) $$Pigment\;concentration=\;\frac{CE\times V}{m}$$

where *CE* is the pigment concentration in the extract (μg·mL^−1^), *V* is the extraction volume (mL), and *m* is the shoot biomass (g FW).

##### Primary Metabolism: Total Soluble and Insoluble Sugars

Total soluble sugars were quantified from 50 mg of tissue homogenized in 1.5 mL of 80% (*v*/*v*) ethanol (homogenizer settings as in lipid peroxidation). Homogenates were diluted in 8.5 mL of 80% (*v*/*v*) ethanol and incubated for 1 h at 80 °C. Then, samples were cooled on ice (10 min), vortex-mixed (15 s), and centrifuged at 10,000× *g* for 10 min at 4 °C. Supernatant aliquots of 30 μL were mixed with 750 μL of 9.8 mM anthrone dissolved in 93% (*v*/*v*) sulfuric acid. The remaining supernatant was discarded, and the pellet was maintained on ice for insoluble sugars quantification. Ethanol served as the blank, and glucose standards prepared with 80% (*v*/*v*) ethanol were used to generate the standard curve. Tubes were incubated at 100 °C for 10 min, cooled on ice (15 min), and vortex-mixed (5 s), before measuring absorbance at 625 nm. For insoluble sugars, 5 mL of 30% (*v*/*v*) perchloric acid was added to each pellet, vortex-mixed for 5 s, and incubated at 60 °C for 1 h. Perchloric acid served as the blank, and glucose standards were prepared in the same solvent. After incubation, the procedure used for soluble sugars was followed, using the respective blank. Final soluble and insoluble sugar contents were calculated from the glucose standard curve and expressed as Glucose Equivalents (GE) (mg GE·g^−1^ FW).

##### Gene Expression Analysis

To investigate the activation of salicylic acid (SA) and jasmonic acid (JA) signaling pathways in kiwifruit under the different treatments applied, the expression of defense-related genes was quantified. The SA-associated genes included *pathogenesis-related protein 1* (*PR1*), a canonical marker of systemic acquired resistance (SAR), *thaumatin-like protein* (*PR5*), and *NIM-interacting protein 2* (*NIMIN2*). The JA pathway was represented by *lipoxygenase 1* (*LOX1*) and *jasmonoyl-isoleucine-12-hydrolase* (*JIH*). Primer sequences and annealing temperatures are listed in Table 3.

Total RNA was extracted using the Plant/Fungi Total RNA Purification Kit (Norgen Biotek Corp., Thorold, ON, Canada), following the manufacturer’s protocol. RNA concentration and purity were evaluated with a Multiskan SkyHigh UV-Vis spectrophotometer (Thermo Scientific, Waltham, MA, USA). Samples were further treated with DNase I Kit (Thermo Scientific^®^) to eliminate residual DNA. cDNA was generated from 1 μg of DNase-treated RNA using the iScript cDNA Synthesis Kit (Bio-Rad^®^, Hercules, CA, USA) in a T100 thermal cycler (Bio-Rad^®^). The resulting cDNA was diluted 1:10 in nuclease-free water and stored at −20 °C.

Reverse transcription quantitative PCR (RT-qPCR) reactions (10 μL) contained 0.3 μL of 10 μM of each forward and reverse primer, 5 μL 2× iQ SYBR Green Supermix (Bio-Rad^®^), and 1 μL cDNA and 3.4 μL of ultrapure water. Four technical replicates were run per biological sample in 96-well Hard-Shell^®^ plates (Bio-Rad^®^) on a CFX96 system (Bio-Rad^®^). Cycling conditions were: 95 °C for 3 min, followed by 40 cycles of 95 °C for 10 s, primer-specific annealing temperatures (Table 3), and 72 °C for 30 s. A melting-curve analysis confirmed amplicon specificity. Relative expression was calculated using the ΔΔC_q_ method, where *Actin* (*ACT*) and *protein phosphatase 2A* (*PP2A*) were used as reference genes [78], and relative expression values are presented as fold change (2^−ΔΔCq^) using the ‘Mock’ treatment as the calibrator.

**Table 3:** Forward (F) and reverse (R) primer sequences, together with their respective annealing temperatures (T_ann_), for all reference (RG) and target (TG) genes included in the RT-qPCR analysis.

Gene	Name		Primer Sequence (5′-3′)	T_ann_ (°C)	Reference
*ACT* (RG)	*Actin*	F	CCAAGGCCAACAGAGAGAAG	56.0	[79]
		R	GACGGAGGATAGCATGAGGA		
*PP2A* (RG)	*Protein phosphatase 2A*	F	GCAGCACATAATTCCACAGG	55.2	[80]
		R	TTTCTGAGCCCATAACAGGAG		
*PR1*	*Pathogenesis-related protein 1*	F	GCCCCCGGTAAGGTTTGT	58.1	[57]
		R	CGAACCAAGACCCACTATTGC		
*PR5*	*Thaumatin-like protein*	F	CGATGGAATTTAGCCCTACG	55.6	[59]
		R	ATTTCCGGAGTTGCAACAGT		
*NIMIN2*	*NIM-interacting protein 2*	F	GAGGGTGGGGGAAGATAA	47.6	[81]
		R	CGTTACCTTTCTCGAAGT		
*LOX1*	*Lipoxygenase 1*	F	GTTAGAGGGGTGGTGACTCT	53.5	[21]
		R	CTTTAGCACTGCTTGGTTGC		
*JIH*	*Jasmonoyl-isoleucine-12-hydrolase*	F	CGGCTCTGATCTGGTTTTTC	55.8	[60]
		R	CCTCATGCTCTCACTCAACG		

### 4.4. CAR Antimicrobial Activity: In Vitro Liquid- and Vapor-Phase Assays

The antimicrobial activity of CAR against Psa was evaluated in *in vitro* trials using liquid- and vapor-phase assays. In both assays, CAR was tested at 0.1% (*w*/*v*), corresponding to the concentration used *in planta*. For the liquid-phase assay, a Psa suspension was prepared as described in Section 4.3.2, except that cells were suspended in LB broth. The suspension was then inoculated in LB broth (final OD_600_ ≈ 0.01) containing CAR emulsion (0.1% *w*/*v* CAR, 2% *v*/*v* Tween20^®^; ‘CAR’ treatment). Control solutions included one without Tween20^®^ (‘LB’) and one containing Tween20^®^ but no CAR (‘Control’). Corresponding blanks without Psa suspension were also prepared. Aliquots of 1.8 mL were transferred to microtubes (*n* = 4) and incubated for 32 h at 28 °C, 180 rpm, in the dark. Viable cells were quantified after 8 h and 32 h by serial dilution and plating on NSA, followed by incubation for 72 h at 28 °C in the dark. Plates containing 30–300 colonies were counted to determine CFU·mL^−1^.

For the vapor-phase assay, the inverted dish method was used [82]. A fresh Psa suspension was prepared as described in Section 4.3.2, adjusted to OD_600_ = 0.10 (1–2 × 10^7^ CFU·mL^−1^), and diluted with Ringer’s solution to 1–2 × 10^4^ CFU·mL^−1^. An aliquot of 100 μL was spread onto 90 mm Petri dishes containing 20 mL NSA (≈5 mm layer; 44.5 cm^3^ free headspace). A sterile 80 mm filter-paper disk was placed in the lid, onto which 500 μL of the CAR emulsion (0.1% *w*/*v* CAR, 2% *v*/*v* Tween20^®^) were applied (‘CAR’ treatment). Plates were immediately inverted and sealed with parafilm. Controls consisted of sterile H_2_O (‘NSA’ treatment) and 2% (*v*/*v*) Tween20^®^ without CAR (‘Control’). Four plates per treatment (*n* = 4) were incubated at 28 °C for 48 h in the dark. Bacteria were then resuspended by adding 5 mL LB broth and six 3 mm glass beads, followed by shaking at 150 rpm for 3 min. Eight 10-fold serial dilutions were prepared in LB broth, and 10-μL drops from the last five dilutions were plated on NSA using the drop-plate method [83]. After incubation at 28 °C for 48 h, colonies were counted to determine viable cell density (CFUs·plate^−1^).

### 4.5. Statistical Analysis

Data analysis was performed using IBM SPSS Statistics (version 28.0.1.0; IBM Corp., Armonk, NY, USA). Homoscedasticity among treatments was verified using Levene’s test, and the effect of the treatments was assessed using one-way ANOVA (treatment factor), followed by Tukey’s post hoc test (α = 0.05). If homogeneity of variance could not be assumed (*p* < 0.05), the data were analyzed using the nonparametric Kruskal–Wallis H test for independent samples. Variables analyzed using the Kruskal–Wallis test are marked with ^†^ in Table 2. In addition, CFU counts from the vapor-phase assay (Figure 5b) were also analyzed using the same nonparametric test. Regarding gene expression, statistical analyses were performed on ΔCq values, whereas fold-change (2^−ΔΔCq^) values are presented for interpretative clarity.

## 5. Conclusions

This study highlights the strong potential of *Elettaria cardamomum* essential oil as a curative treatment against the Gram-negative pathogen Psa and, for the first time, provides insight into its underlying mechanisms of action. When applied to kiwifruit plants at an early symptomatic stage, CAR reduced KBC symptoms and demonstrated a direct, though transitory, activity against Psa. Moreover, this study explored whether CAR could activate host defense mechanisms that are crucial for enhanced protection against subsequent infections, but no plant-eliciting effect was observed within the scope of our biochemical and molecular analyses. Although no defense-related effects were detected within the evaluated window, this does not rule out the possibility that CAR may influence additional pathways or time points beyond the scope of this study. Importantly, CAR did not suppress the SAR-related transcriptional responses typically triggered by Psa infection, as evidenced by the similar upregulation of *PR1* and *PR5* in inoculated plants regardless of CAR treatment, suggesting that this EO does not interfere with the SA-dependent defense signaling pathways examined in this study. A liquid-phase *in vitro* assay suggested a transient inhibition of Psa growth during exposure to CAR, which may partly account for the reduction in KBC symptom severity observed in CAR-treated plants. This anti-Psa effect appears to depend on the full direct liquid contact between CAR and bacterial cells, as indicated by the lack of inhibition of Psa solely exposed to the CAR’s vapor phase. Among the CAR’s constituents, 1,8-cineole and α-terpinyl acetate are highlighted as priority candidates for further investigation, given their predominance in the oil’s composition and their membrane-targeting properties previously reported against bacterial pathogens. Nevertheless, further studies are necessary to verify possible interactions among CAR constituents, and such investigations should be extended to the most abundant monoterpenes in the present CAR, whose collective contribution to the observed antimicrobial activity should not be overlooked. Moreover, field trials are required, preferably under different edaphoclimatic conditions and in orchards at distinct plant developmental stages, to fully assess the CAR’s curative capacity in counteracting Psa infection. Altogether, this will deepen the knowledge of the underlying modes of action and likely support the development of novel formulations based on CAR’s composition. Such progress is particularly relevant given that the plant–pathogen–EO interactions remain poorly understood, and these formulations hold promise for deploying effective curative methods against KBC using natural bioactive molecules.

## Figures and Tables

**Table 1:** Volatile headspace profile of cardamom essential oil obtained by HS-SPME-GC-MS. The table presents the compounds identified in the headspace fraction, their retention times (RT), the literature retention index ranges [RI (lit.)], experimental retention indices [RI (exp.)], chemical classes, and relative peak areas, expressed as percentage of the total chromatographic area. Experimental RI values were calculated using an n-alkane homologous series and compared with the literature values reported for polar GC columns. Compounds were identified by comparison of mass spectra with spectral libraries and supported by agreement between the experimental and literature RI values.

Compound	RT (min)	RI (lit.)	RI (exp.)	Chemical Class	Relative Abundance (%)
α-Pinene	7.974	989–1020	993	Monoterpene hydrocarbon	0.620
β-Thujene	8.211	978–1050	1002	Monoterpene hydrocarbon	0.988
β-Pinene	10.685	1055–1110	1068	Monoterpene hydrocarbon	0.468
(+)-Sabinene	11.404	1074–1140	1087	Monoterpene hydrocarbon	1.77
3-Carene	12.653	1105–1130	1115	Monoterpene hydrocarbon	0.137
β-Myrcene	13.722	1130–1170	1136	Monoterpene hydrocarbon	1.65
α-Terpinene	14.239	1155–1195	1146	Monoterpene hydrocarbon	0.310
D-Limonene	15.209	1152–1210	1166	Monoterpene hydrocarbon	5.73
1,8-Cineole	15.634	1167–1253	1174	Oxygenated monoterpene; monoterpene ether	35.6
γ-Terpinene	17.827	1210–1260	1215	Monoterpene hydrocarbon	0.524
p-Cymene	19.316	1232–1285	1241	Monoterpene hydrocarbon; aromatic monoterpene	3.92
α-Terpinolene	20.009	1235–1285	1252	Monoterpene hydrocarbon	0.373
Linalool	36.325	1515–1565	1524	Oxygenated monoterpene; monoterpene alcohol	1.30
Linalyl acetate	36.734	1523–1575	1531	Oxygenated monoterpene; monoterpene ester	3.35
α-Bergamotene	37.859	1545–1610	1550	Sesquiterpene hydrocarbon	0.948
β-Elemen	38.105	1540–1615	1554	Sesquiterpene hydrocarbon	0.375
Terpinen-4-ol	39.035	1550–1640	1570	Oxygenated monoterpene; monoterpene alcohol	0.203
α-Terpinyl acetate	44.252	1648–1710	1662	Oxygenated monoterpene; monoterpene ester	32.6
Selinene	44.883	1666–1740	1673	Sesquiterpene hydrocarbon	2.70
β-Bisabolene	45.862	1678–1745	1691	Sesquiterpene hydrocarbon	0.121
Nerol acetate	46.242	1680–1730	1697	Oxygenated monoterpene; monoterpene ester	0.0565
γ-Cadinene	47.217	1695–1715	1715	Sesquiterpene hydrocarbon	4.42
Geranyl acetate	47.892	1710–1740	1727	Oxygenated monoterpene; monoterpene ester	0.267
β-Sesquiphellandrene	48.110	1720–1760	1731	Sesquiterpene hydrocarbon	0.126
Curcumene	48.413	1727–1790	1737	Sesquiterpene hydrocarbon	0.198
α-Cadinene	49.034	1720–1815	1748	Sesquiterpene hydrocarbon	0.324
Calamenene	51.138	1770–1820	1786	Sesquiterpene hydrocarbon; aromatic sesquiterpene	0.506
α-epi-Cadinol	67.726	2120–2170	2161	Oxygenated sesquiterpene; sesquiterpene alcohol	0.283
Eugenol	68.059	2140–2213	2162	Phenylpropanoid; phenolic compound	0.119
Total identified composition: 99.99%					

**Table 2:** Concentrations of total phenolics, total flavonoids, anthocyanins, carotenoids, chlorophylls a and b, soluble sugars and insoluble sugars in *Actinidia chinensis* var. *deliciosa* ‘Hayward’ plants mock inoculated (‘Mock’), or artificially inoculated with *Pseudomonas syringae* pv. *actinidiae* (Psa) and treated with emulsion solution without (‘Psa’) or with (‘Psa+CAR’) cardamom essential oil. Results are presented as the mean ± standard error of the mean (*n* = 3; each replicate consisting of a pooled sample of three plants), at 1 and 14 days after treatment (DAT) with the essential oil. ^†^ Denotes results analyzed nonparametrically using the Kruskal–Wallis H test.

**Treatment**	**Total phenolics** **(μg GAE·g^−1^ FW)**		**Total flavonoids** **(mg CE·g^−1^ FW)**		**Anthocyanins** **(A·g^−1^ FW)**		**Carotenoids** **(µg·g^−1^ FW)**	
	**1 DAT**	**14 DAT**	**1 DAT**	**14 DAT**	**1 DAT**	**14 DAT**	**1 DAT**	**14 DAT**
Mock	6.52 ± 0.11	9.69 ± 1.92	3.03 ± 0.24	3.65 ± 0.72	0.41 ± 0.06	0.54 ± 0.04	190 ± 30	85 ± 8
Psa	9.03 ± 0.48	13.11 ± 3.32	5.85 ± 0.64	7.89 ± 2.51	0.30 ± 0.03	0.58 ± 0.09	236 ± 12	97 ± 18
Psa+CAR	7.91 ± 1.17	11.89 ± 0.48	6.18 ± 1.17	6.86 ± 0.45	0.24 ± 0.02	0.49 ± 0.02	224 ± 5	104 ± 13
*p*-value	0.131	0.393 ^†^	0.055	0.193	0.065	0.733 ^†^	0.278	0.838
**Treatment**	**Chlorophylls a** **(µg·g^−1^ FW)**		**Chlorophylls b** **(µg·g^−1^ FW)**		**Soluble sugars** **(mg GE·g^−1^ FW)**		**Insoluble sugars** **(mg GE·g^−1^ FW)**	
	**1 DAT**	**14 DAT**	**1 DAT**	**14 DAT**	**1 DAT**	**14 DAT**	**1 DAT**	**14 DAT**
Mock	529 ± 82	222 ± 30	236 ± 40	105 ± 16	31.9 ± 2.8	32.3 ± 1.6	18.4 ± 1.4	13.2 ± 2.8
Psa	621 ± 21	241 ± 41	261 ± 2	107 ± 16	44.0 ± 4.6	37.4 ± 4.7	21.3 ± 4.7	16.5 ± 5.5
Psa+CAR	656 ± 25	253 ± 31	273 ± 9	113 ± 16	38.5 ± 1.7	42.5 ± 2.1	24.7 ± 3.1	12.3 ± 2.7
*p*-value	0.271	0.952	0.421 ^†^	0.963	0.099	0.144	0.455	0.743

Abbreviations: A = absorbances; CE = catechin equivalents; FW = fresh weight; GAE = gallic acid equivalents; GE = glucose equivalents.

## Data Availability

The original contributions presented in this study are included in the article. Further inquiries can be directed to the corresponding author.
